# Supplementary material for: Assessing the Safety of Mechanically Fibrillated Cellulose Nanofibers (fib-CNF) via Toxicity Tests on Mice: Single Intratracheal Administration and 28 Days’ Oral Intake
Source: Toxics. 2024 Feb 1;12(2):121. doi: 10.3390/toxics12020121 (PMC10893282; doi:10.3390/toxics12020121)
Supplement: Supplementary file 1 [file toxics-12-00121-s001.zip › toxics-2759942-supplementary.pdf]

Supplementary Materials

Assessing the Safety of Mechanically Fibrillated Cellulose Nanofibers (fib-CNF) via Toxicity Tests on Mice: Single Intratracheal Administration and 28 Days' Oral Intake

Yoshihiro Yamashita [1,*], Akinori Tokunaga [2,6], Koji Aoki [3,6], Tamotsu Ishizuka [4,6], Hideyuki Uematsu [5,6], Hiroaki Sakamoto [5,6], Satoshi Fujita [5,6] and Shuichi Tanoue [1,6]

[1] Research Center for Fibers and Materials, University of Fukui, 3-9-1 Bunkyo, Fukui 910-8507, Japan; tanoue@u-fukui.ac.jp
[2] Life Science Research Laboratory, University of Fukui, 23-3, Matsuoka Shimoaizuki, Eiheiji-cho, Fukui 910-1193, Japan; aktoku@u-fukui.ac.jp
[3] Department of Pharmacology, Faculty of Medicine, University of Fukui, 23-3, Matsuoka Shimoaizuki, Eiheiji-cho, Fukui 910-1193, Japan; aokik@u-fukui.ac.jp
[4] Third Department of Internal Medicine, Faculty of Medicine, University of Fukui, 23-3, Matsuoka Shimoaizuki, Eiheiji-cho, Fukui 910-1193, Japan; tamotsui@u-fukui.ac.jp
[5] Department of Frontier Fiber Technology and Science, Graduate School of Engineering, University of Fukui, 3-9-1 Bunkyo, Fukui 910-8507, Japan; uematsu@matse.u-fukui.ac.jp (H.U.); hi-saka@u-fukui.ac.jp (H.S.); fujitas@u-fukui.ac.jp (S.F.)
[6] Organization for Life Science Advancement Programs, University of Fukui. 3-9-1 Bunkyo, Fukui 910-8507
* Correspondence: yama-yo@u-fukui.ac.jp; Tel.: +81-50-3633-8582

Table S1. Raw data for Figure 2. Body weight of mice 3 days after a single intratracheal administration of fib-CNF; Orange: control (100 μL of distilled water only); Blue: fib-CNF (0.2 mg/100 μL).

Sex	Test article	Dose (mg/body)	Animal number	Mode of death(day)	Body weight (g)	Lungs (g)
Male	Control	0	01M01		26.3	0.134
			01M02		28.2	0.146
			01M03		26.7	0.132
			01M04		25.9	0.140
			01M05		27.8	0.140
			Mean		27.0	0.138
			S.D.		1.0	0.006
	CNF 「BiNFi-s」 FMa-10002	0.2	02M01		26.2	0.189
			02M02		25.6	0.198
			02M03		25.4	0.201
			02M04		26.1	0.200
			02M05		25.3	0.182
			Mean		25.7	0.194
			S.D.		0.4	0.008

Table S2. Raw data for Figure 2. Body weight of mice 28 days after a single intratracheal administration of fib-CNF; Orange: control (100 μL of distilled water only); Blue: fib-CNF (0.2 mg/100 μL).

Sex	Test article	Dose (mg/body)	Animal number	Mode of death(day)	Body weight (g)	Lungs (g)
Male	Control-2	0	03M01		29.5	0.141
			03M02		28.1	0.137
			03M03		27.8	0.135
			03M04		28.2	0.138
			03M05		29.2	0.156
			Mean		28.6	0.141
			S.D.		0.7	0.008
	CNF 「BiNFi-s」 FMa-10002	0.2	04M01		28.8	0.164
			04M02		29.3	0.177
			04M03		27.7	0.170
			04M04		28.8	0.199
			04M05		28.1	0.177
			Mean		28.5	0.177
			S.D.		0.6	0.013

Table S3. Raw data for Table 4**.** Hematology parameters of each group (mean ± standard deviation) measured 3 days post-administration.

Sex	Test article	Dose (mg/body)	Animal number	Mode of death(day)	RBC (x10^4/μL)	HGB (g/dL)	HCT (%)	MCV (fL)	MCH (pg)	MCHC (g/dL)	PLT (x10^4/μL)	Reticulo-cytes (x10^4/μL)
Male	Control	0	01M01		912	13.3	40.9	44.8	14.6	32.5	133.6	37.03
			01M02		965	13.9	43.8	45.4	14.4	31.7	157.4	41.01
			01M03		898	13.4	43.4	48.3	14.9	30.9	126.1	58.64
			01M04		-a	-a	-a	-a	-a	-a	-a	-a
			01M05		931	13.5	41.9	45.0	14.5	32.2	153.2	40.87
			Mean		927	13.5	42.5	45.9	14.6	31.8	142.6	44.39
			S.D.		29	0.3	1.3	1.6	0.2	0.7	15.1	9.68
	CNF 「BiNFi-s」 FMa-10002	0.2	02M01		962	14.0	42.8	44.5	14.6	32.7	161.7	16.45
			02M02		895	13.5	42.1	47.0	15.1	32.1	57.2	29.80
			02M03		904	13.4	42.7	47.2	14.8	31.4	160.5	35.17
			02M04		913	13.6	42.9	47.0	14.9	31.7	161.3	37.71
			02M05		944	13.5	42.4	44.9	14.3	31.8	154.2	28.98
			Mean		924	13.6	42.6	46.1	14.7	31.9	139.0	29.62
			S.D.		28	0.2	0.3	1.3	0.3	0.5	45.8	8.22

a : Data omitted from the analysis for coagulation.

Sex	Test article	Dose (mg/body)	Animal number	Mode of death(day)	WBC (x10^2/μL)	Leukocytes, differential: Lympho-cytes (x10^2/μL)	Neutro-phils (x10^2/μL)	Eosino-phils (x10^2/μL)	Mono-cytes (x10^2/μL)	Baso-phils (x10^2/μL)
Male	Control	0	01M01		46.9	41.6	3.1	1.1	1.1	0.0
			01M02		54.5	47.7	4.0	1.1	1.7	0.0
			01M03		29.2	25.0	2.5	0.7	1.0	0.0
			01M04		-a	-a	-a	-a	-a	-a
			01M05		42.0	36.4	3.2	1.2	1.2	0.0
			Mean		43.2	37.7	3.2	1.0	1.3	0.0
			S.D.		10.6	9.6	0.6	0.2	0.3	0.0
	CNF 「BiNFi-s」 FMa-10002	0.2	02M01		59.6	48.1	5.0	3.4	3.1	0.0
			02M02		45.8	38.2	3.6	2.2	1.8	0.0
			02M03		25.0	21.5	1.6	1.1	0.8	0.0
			02M04		49.6	42.2	2.7	3.0	1.7	0.0
			02M05		29.2	23.7	3.1	1.5	0.9	0.0
			Mean		41.8	34.7	3.2	2.2	1.7	0.0
			S.D.		14.4	11.7	1.2	1.0	0.9	0.0

a : Data omitted from the analysis for coagulation.

Table S4. Raw data for Table 5**.** Hematology parameters of each group (mean ± standard deviation) measured 28 days post-administration.

Sex	Test article	Dose (mg/body)	Animal number	Mode of death(day)	RBC (x10^4/μL)	HGB (g/dL)	HCT (%)	MCV (fL)	MCH (pg)	MCHC (g/dL)	PLT (x10^4/μL)	Reticulo-cytes (x10^4/μL)
Male	Control-2	0	03M01		997	14.6	45.2	45.3	14.6	32.3	139.1	47.76
			03M02		985	14.2	44.6	45.3	14.4	31.8	156.1	41.67
			03M03		953	13.9	43.8	46.0	14.6	31.7	151.4	47.65
			03M04		974	14.3	44.8	46.0	14.7	31.9	157.4	46.85
			03M05		972	13.6	42.1	43.3	14.0	32.3	121.4	49.77
			Mean		976	14.1	44.1	45.2	14.5	32.0	145.1	46.74
			S.D.		16	0.4	1.2	1.1	0.3	0.3	15.1	3.03
	CNF 「BiNFi-s」 FMa-10002	0.2	04M01		954	14.2	44.3	46.4	14.9	32.1	112.4	42.55
			04M02		986	14.3	44.7	45.3	14.5	32.0	149.6	41.02
			04M03		994	13.9	45.1	45.4	14.0	30.8	164.5	39.56
			04M04		931	13.9	43.6	46.8	14.9	31.9	111.7	50.65
			04M05		1007	14.6	45.9	45.6	14.5	31.8	166.4	41.79
			Mean		974	14.2	44.7	45.9	14.6	31.7	140.9	43.11
			S.D.		31	0.3	0.9	0.7	0.4	0.5	27.1	4.36

Sex	Test article	Dose (mg/body)	Animal number	Mode of death(day)	WBC (x10^2/μL)	Leukocytes, differential: Lympho-cytes (x10^2/μL)	Neutro-phils (x10^2/μL)	Eosino-phils (x10^2/μL)	Mono-cytes (x10^2/μL)	Baso-phils (x10^2/μL)
Male	Control-2	0	03M01		41.7	34.9	4.1	0.7	2.0	0.0
			03M02		89.1	75.7	7.9	1.5	4.0	0.0
			03M03		74.7	64.3	7.0	1.0	2.4	0.0
			03M04		80.9	71.2	5.9	1.0	2.8	0.0
			03M05		62.1	53.7	4.7	0.6	3.1	0.0
			Mean		69.7	60.0	5.9	1.0	2.9	0.0
			S.D.		18.5	16.3	1.6	0.4	0.8	0.0
	CNF 「BiNFi-s」 FMa-10002	0.2	04M01		39.2	34.6	2.9	0.4	1.3	0.0
			04M02		79.9	70.5	5.6	1.0	2.8	0.0
			04M03		106.3	92.7	8.3	1.7	3.6	0.0
			04M04		90.5	80.0	5.8	1.5	3.2	0.0
			04M05		85.9	74.9	7.2	0.9	2.9	0.0
			Mean		80.4	70.5	6.0	1.1	2.8	0.0
			S.D.		25.0	21.7	2.0	0.5	0.9	0.0

Table S5. Raw data for Table 6. Organ weights—group mean values (mean ± S.D.) at 3 days after administration.

Sex	Test article	Dose (mg/body)	Animal number	Mode of death(day)	Body weight (g)	Lungs (g)
Male	Control	0	01M01		26.3	0.134
			01M02		28.2	0.146
			01M03		26.7	0.132
			01M04		25.9	0.140
			01M05		27.8	0.140
			Mean		27.0	0.138
			S.D.		1.0	0.006
	CNF「BiNFi-s」FMa-10002	0.2	02M01		26.2	0.189
			02M02		25.6	0.198
			02M03		25.4	0.201
			02M04		26.1	0.200
			02M05		25.3	0.182
			Mean		25.7	0.194
			S.D.		0.4	0.008

Table S6. Raw data for Table 7. Organ weights—group mean values (mean ± S.D.) at 28 days after administration.

Sex	Test article	Dose (mg/body)	Animal number	Mode of death(day)	Body weight (g)	Lungs (g)
Male	Control-2	0	03M01		29.5	0.141
			03M02		28.1	0.137
			03M03		27.8	0.135
			03M04		28.2	0.138
			03M05		29.2	0.156
			Mean		28.6	0.141
			S.D.		0.7	0.008
	CNF「BiNFi-s」FMa-10002	0.2	04M01		28.8	0.164
			04M02		29.3	0.177
			04M03		27.7	0.170
			04M04		28.8	0.199
			04M05		28.1	0.177
			Mean		28.5	0.177
			S.D.		0.6	0.013

Table S7. Raw data for Table 8. Histopathological evaluation of lung/bronchial alveolar macrophage aggregation in male mice, 3 days post-administration.

Animal number	: 01M01	Sex : Male	Mode of death	: Interim sacrifice	Week(day) of death	: 1	(3)
Test article	: Control		Dose (mg/body)	: 0	Stage of death	: Treatment period	

Microscopic examination

The following organs were not remarkable: Lung/bronchial

Animal number	: 01M02	Sex : Male	Mode of death	: Interim sacrifice	Week(day) of death	: 1	(3)
Test article	: Control		Dose (mg/body)	: 0	Stage of death	: Treatment period	

Microscopic examination

The following organs were not remarkable: Lung/bronchial

Animal number	: 01M03	Sex : Male	Mode of death	: Interim sacrifice	Week(day) of death	: 1	(3)
Test article	: Control		Dose (mg/body)	: 0	Stage of death	: Treatment period	

Microscopic examination

The following organs were not remarkable: Lung/bronchial

Animal number	: 01M04	Sex : Male	Mode of death	: Interim sacrifice	Week(day) of death	: 1	(3)
Test article	: Control		Dose (mg/body)	: 0	Stage of death	: Treatment period	

Microscopic examination

The following organs were not remarkable: Lung/bronchial

Animal number	: 01M05	Sex : Male	Mode of death	: Interim sacrifice	Week(day) of death	: 1	(3)
Test article	: Control		Dose (mg/body)	: 0	Stage of death	: Treatment period	

Microscopic examination

The following organs were not remarkable: Lung/bronchial

Animal number	: 02M01	Sex : Male	Mode of death	: Interim sacrifice	Week(day) of death	: 1	(3)
Test article	: CNF 「BiNFi-s」 FMa-10002		Dose (mg/body)	: 0.2	Stage of death	: Treatment period	

Microscopic examination

Lung/bronchial

Alveolar macrophage aggregation : moderate

Animal number	: 02M02	Sex : Male	Mode of death	: Interim sacrifice	Week(day) of death	: 1	(3)
Test article	: CNF 「BiNFi-s」 FMa-10002		Dose (mg/body)	: 0.2	Stage of death	: Treatment period	

Microscopic examination

Lung/bronchial

Alveolar macrophage aggregation : moderate

Animal number	: 02M03	Sex : Male	Mode of death	: Interim sacrifice	Week(day) of death	: 1	(3)
Test article	: CNF 「BiNFi-s」 FMa-10002		Dose (mg/body)	: 0.2	Stage of death	: Treatment period	

Microscopic examination

Lung/bronchial

Alveolar macrophage aggregation : moderate

Animal number	: 02M04	Sex : Male	Mode of death	: Interim sacrifice	Week(day) of death	: 1	(3)
Test article	: CNF 「BiNFi-s」 FMa-10002		Dose (mg/body)	: 0.2	Stage of death	: Treatment period	

Microscopic examination

Lung/bronchial

Alveolar macrophage aggregation : moderate

Animal number	: 02M05	Sex : Male	Mode of death	: Interim sacrifice	Week(day) of death	: 1	(3)
Test article	: CNF 「BiNFi-s」 FMa-10002		Dose (mg/body)	: 0.2	Stage of death	: Treatment period	

Microscopic examination

Lung/bronchial

Alveolar macrophage aggregation : moderate

Table S8. Raw data for Table 9. Histopathological evaluation of lung/bronchial alveolar macrophage aggregation in male mice, 28 days post-administration.

Animal number	: 03M01	Sex : Male	Mode of death	: Scheduled sacrifice	Week(day) of death	: 5 (28)
Test article	: Control-2		Dose (mg/body)	: 0	Stage of death	: Treatment period

Microscopic examination

The following organs were not remarkable: Lung/bronchial

Animal number	: 03M02	Sex : Male	Mode of death	: Scheduled sacrifice	Week(day) of death	: 5 (28)
Test article	: Control-2		Dose (mg/body)	: 0	Stage of death	: Treatment period

Microscopic examination

The following organs were not remarkable: Lung/bronchial

Animal number	: 03M03	Sex : Male	Mode of death	: Scheduled sacrifice	Week(day) of death	: 5 (28)
Test article	: Control-2		Dose (mg/body)	: 0	Stage of death	: Treatment period

Microscopic examination

The following organs were not remarkable: Lung/bronchial

Animal number	: 03M04	Sex : Male	Mode of death	: Scheduled sacrifice	Week(day) of death	: 5 (28)
Test article	: Control-2		Dose (mg/body)	: 0	Stage of death	: Treatment period

Microscopic examination

The following organs were not remarkable: Lung/bronchial

Animal number	: 03M05	Sex : Male	Mode of death	: Scheduled sacrifice	Week(day) of death	: 5 (28)
Test article	: Control-2		Dose (mg/body)	: 0	Stage of death	: Treatment period

Microscopic examination

The following organs were not remarkable: Lung/bronchial

Animal number	: 04M01	Sex : Male	Mode of death	: Scheduled sacrifice	Week(day) of death	: 5 (28)
Test article	: CNF 「BiNFi-s」 FMa-10002		Dose (mg/body)	: 0.2	Stage of death	: Treatment period

Microscopic examination

Lung/bronchial

Alveolar macrophage aggregation : moderate

Animal number	: 04M02	Sex : Male	Mode of death	: Scheduled sacrifice	Week(day) of death	: 5 (28)
Test article	: CNF 「BiNFi-s」 FMa-10002		Dose (mg/body)	: 0.2	Stage of death	: Treatment period

Microscopic examination

Lung/bronchial

Alveolar macrophage aggregation : moderate

Animal number	: 04M03	Sex : Male	Mode of death	: Scheduled sacrifice	Week(day) of death	: 5 (28)
Test article	: CNF 「BiNFi-s」 FMa-10002		Dose (mg/body)	: 0.2	Stage of death	: Treatment period

Microscopic examination

Lung/bronchial

Alveolar macrophage aggregation : moderate

Animal number	: 04M04	Sex : Male	Mode of death	: Scheduled sacrifice	Week(day) of death	: 5 (28)
Test article	: CNF 「BiNFi-s」 FMa-10002		Dose (mg/body)	: 0.2	Stage of death	: Treatment period

Microscopic examination

Lung/bronchial

Alveolar macrophage aggregation : moderate

Animal number	: 04M05	Sex : Male	Mode of death	: Scheduled sacrifice	Week(day) of death	: 5 (28)
Test article	: CNF 「BiNFi-s」 FMa-10002		Dose (mg/body)	: 0.2	Stage of death	: Treatment period

Microscopic examination

Lung/bronchial

Alveolar macrophage aggregation : moderate

Table S9. Raw data for Figure 5. Body weight of mice after oral administration of fib-CNF for 28 days of control and forced administration groups of 400 mg/kg/day of fib-CNF. No significant difference was observed compared to the control group.

Sex	Test article	Dose (mg/kg/day)	Animal number	Mode of death(day)	Initial B.W.	Day 7	14	21	28	29 a
Male	Control	0	01N01		24.2	23.9	25.0	24.3	24.9	21.0
			01N02		24.5	24.6	25.1	25.5	26.0	22.1
			01N03		22.9	23.8	24.3	25.2	25.4	21.5
			01N04	MS (13)	22.9	23.0				
			01N05		23.6	24.7	24.2	24.8	25.0	21.2
			01N06		23.6	24.2	25.1	25.3	25.9	21.9
			01N07		23.7	24.1	23.8	24.2	24.7	21.0
			01N08		24.3	25.4	24.4	24.4	24.8	21.0
			01N09		24.0	24.5	24.7	25.4	25.5	21.9
			01N10		23.4	24.6	24.5	25.1	25.5	21.6
			Mean		23.7	24.3	24.6	24.9	25.3	21.5
			S.D.		0.6	0.6	0.4	0.5	0.5	0.4
	CNF「BiNFi-s」FMa-10002	400	02N01		24.4	24.7	25.9	26.0	27.0	22.9
			02N02		23.6	23.6	24.3	24.5	24.6	21.1
			02N03		24.1	24.4	25.5	26.1	27.0	22.9
			02N04		23.7	23.8	24.2	24.7	24.9	21.1
			02N05		24.6	23.7	24.4	24.8	25.7	21.6
			02N06		24.0	24.6	24.3	25.3	25.3	21.8
			02N07		23.4	24.9	24.7	25.5	26.2	22.0
			02N08		23.8	24.1	24.7	24.5	25.2	21.2
			021N09		24.4	24.1	24.6	25.1	25.4	21.7
			02N10		23.2	23.7	24.5	24.4	25.0	20.7
			Mean		23.9	24.2	24.7	25.1	25.6	21.7
			S.D.		0.5	0.5	0.6	0.6	0.8	0.7

MS : Moribund sacrifice
a : The value presented was obtained after the animal was fasted overnight.

Table S10. Raw data for Table 10. Hematology—group mean values (mean ± S.D.).

Sex	Test article	Dose (mg/kg/day)	Animal number	Mode of death(day)	RBC	HGB	HCT	MCV	MCH	MCHC	PLT	Reticulo-cytes
					($x10^4/\mu L$)	(g/dL)	(%)	(fL)	(pg)	(g/dL)	($x10^4/\mu L$)	($x10^4/\mu L$)
Male	Control	0	01N01		947	14.1	42.4	44.8	14.9	33.3	154.6	45.83
			01N02		970	14.6	43.5	44.8	15.1	33.6	158.5	50.83
			01N03		974	14.3	43.2	44.4	14.7	33.1	150.6	46.56
			01N05		994	14.4	43.9	44.2	14.5	32.8	154.9	47.71
			01N06		1003	14.9	44.3	44.2	14.9	33.6	151.9	49.25
			Mean		978	14.5	43.5	44.5	14.8	33.3	154.1	48.04
			S.D.		22	0.3	0.7	0.3	0.2	0.3	3.1	2.03
	CNF「BiNFi-s」FMa-10002	400	02N01		974	14.7	44.0	45.2	15.1	33.4	145.5	47.82
			02N02		996	14.8	44.3	44.5	14.9	33.4	148.5	47.91
			02N03		968	14.4	43.3	44.7	14.9	33.3	148.5	49.46
			02N04		984	14.4	43.6	44.3	14.6	33.0	150.1	45.56
			02N05		942	14.0	42.4	45.0	14.9	33.0	137.7	47.67
			Mean		973	14.5	43.5	44.7	14.9	33.2	146.1	47.68
			S.D.		20	0.3	0.7	0.4	0.2	0.2	5.0	1.39

Sex	Test article	Dose (mg/kg/day)	Animal number	Mode of death(day)	WBC	Leukocytes, differential				
						Lympho-cytes	Neutro-phils	Eosino-phils	Baso-phils	Mono-cytes
					($x10^2/\mu L$)	($x10^2/\mu L$)	($x10^2/\mu L$)	($x10^2/\mu L$)	($x10^2/\mu L$)	($x10^2/\mu L$)
Male	Control	0	01N01		19.7	15.7	3.5	0.0	0.0	0.5
			01N02		21.2	17.5	3.0	0.1	0.0	0.6
			01N03		14.4	11.0	3.0	0.0	0.0	0.4
			01N05		11.8	10.4	1.0	0.0	0.0	0.4
			01N06		8.5	-a	-a	-a	-a	-a
			Mean		15.1	13.7	2.6	0.0	0.0	0.5
			S.D.		5.3	3.5	1.1	0.1	0.0	0.1
	CNF「BiNFi-s」FMa-10002	400	02N01		21.9	18.6	2.7	0.0	0.0	0.6
			02N02		11.3	9.1	2.0	0.0	0.0	0.2
			02N03		11.3	9.5	1.6	0.0	0.0	0.2
			02N04		15.3	12.6	2.1	0.0	0.0	0.6
			02N05		14.4	12.6	1.3	0.0	0.0	0.5
			Mean		14.8	12.5	1.9	0.0	0.0	0.4
			S.D.		4.3	3.8	0.5	0.0	0.0	0.2

a : Excluded due to unreliable data.

Table S11. Raw data for 3.3.5: Histopathological Examination

Animal number : 01N01 Sex : Male Mode of death : Scheduled sacrifice Week(day) of death : 5 (29)
Test article : Control Dose (mg/kg/day) : 0 Stage of death : Treatment period
Macroscopic examination
All organs: Not remarkable

Animal number : 01N02 Sex : Male Mode of death : Scheduled sacrifice Week(day) of death : 5 (29)
Test article : Control Dose (mg/kg/day) : 0 Stage of death : Treatment period
Macroscopic examination
All organs: Not remarkable

Animal number : 01N03 Sex : Male Mode of death : Scheduled sacrifice Week(day) of death : 5 (29)
Test article : Control Dose (mg/kg/day) : 0 Stage of death : Treatment period
Macroscopic examination
All organs: Not remarkable

Animal number : 01N04 Sex : Male Mode of death : Moribund sacrifice Week(day) of death : 2 (13)
Test article : Control Dose (mg/kg/day) : 0 Stage of death : Treatment period
Macroscopic examination
Stomach
Contained material, Black
Small intestine
Contained material, Black
Other organs: Not remarkable

Animal number : 01N05 Sex : Male Mode of death : Scheduled sacrifice Week(day) of death : 5 (29)
Test article : Control Dose (mg/kg/day) : 0 Stage of death : Treatment period
Macroscopic examination
All organs: Not remarkable

Animal number : 01N06 Sex : Male Mode of death : Scheduled sacrifice Week(day) of death : 5 (29)
Test article : Control Dose (mg/kg/day) : 0 Stage of death : Treatment period
Macroscopic examination
All organs: Not remarkable

Animal number : 01N07 Sex : Male Mode of death : Scheduled sacrifice Week(day) of death : 5 (29)
Test article : Control Dose (mg/kg/day) : 0 Stage of death : Treatment period
Macroscopic examination
All organs: Not remarkable

Animal number : 01N08 Sex : Male Mode of death : Scheduled sacrifice Week(day) of death : 5 (29)
Test article : Control Dose (mg/kg/day) : 0 Stage of death : Treatment period
Macroscopic examination
All organs: Not remarkable

Animal number : 01N09 Sex : Male Mode of death : Scheduled sacrifice Week(day) of death : 5 (29)
Test article : Control Dose (mg/kg/day) : 0 Stage of death : Treatment period
Macroscopic examination
All organs: Not remarkable

Animal number : 01N10 Sex : Male Mode of death : Scheduled sacrifice Week(day) of death : 5 (29)
Test article : Control Dose (mg/kg/day) : 0 Stage of death : Treatment period
Macroscopic examination
All organs: Not remarkable

Animal number : 02N01 Sex : Male Mode of death : Scheduled sacrifice Week(day) of death : 5 (29)
Test article : CNF「BiNFi-s」FMa-10002 Dose (mg/kg/day) : 400 Stage of death : Treatment period
Macroscopic examination
All organs: Not remarkable

Animal number : 02N02 Sex : Male Mode of death : Scheduled sacrifice Week(day) of death : 5 (29)
Test article : CNF「BiNFi-s」FMa-10002 Dose (mg/kg/day) : 400 Stage of death : Treatment period
Macroscopic examination
All organs: Not remarkable

Animal number : 02N03 Sex : Male Mode of death : Scheduled sacrifice Week(day) of death : 5 (29)
Test article : CNF「BiNFi-s」FMa-10002 Dose (mg/kg/day) : 400 Stage of death : Treatment period
Macroscopic examination
All organs: Not remarkable

Animal number : 02N04 Sex : Male Mode of death : Scheduled sacrifice Week(day) of death : 5 (29)
Test article : CNF「BiNFi-s」FMa-10002 Dose (mg/kg/day) : 400 Stage of death : Treatment period
Microscopic examination
The following organs were not remarkable: Stomach, Duodenum, Jejunum, Ileum, Cecum, Colon, Rectum

Animal number : 02N05 Sex : Male Mode of death : Scheduled sacrifice Week(day) of death : 5 (29)
Test article : CNF「BiNFi-s」FMa-10002 Dose (mg/kg/day) : 400 Stage of death : Treatment period
Microscopic examination
The following organs were not remarkable: Stomach, Duodenum, Jejunum, Ileum, Cecum, Colon, Rectum

Animal number : 02N06 Sex : Male Mode of death : Scheduled sacrifice Week(day) of death : 5 (29)
Test article : CNF「BiNFi-s」FMa-10002 Dose (mg/kg/day) : 400 Stage of death : Treatment period
Microscopic examination
The following organs were not remarkable: Stomach, Duodenum, Jejunum, Ileum, Cecum, Colon, Rectum

Animal number 02N07 Sex : Male Mode of death : Scheduled sacrifice Week(day) of death : 5 (29)
Test article : CNF「BiNFi-s」FMa-10002 Dose (mg/kg/day) : 400 Stage of death : Treatment period
Microscopic examination
The following organs were not remarkable: Stomach, Duodenum, Jejunum, Ileum, Cecum, Colon, Rectum

Animal number 02N08 Sex : Male Mode of death : Scheduled sacrifice Week(day) of death : 5 (29)
Test article : CNF「BiNFi-s」FMa-10002 Dose (mg/kg/day) : 400 Stage of death : Treatment period
Microscopic examination
The following organs were not remarkable: Stomach, Duodenum, Jejunum, Ileum, Cecum, Colon, Rectum

Animal number 02N09 Sex : Male Mode of death : Scheduled sacrifice Week(day) of death : 5 (29)
Test article : CNF「BiNFi-s」FMa-10002 Dose (mg/kg/day) : 400 Stage of death : Treatment period
Microscopic examination
The following organs were not remarkable: Stomach, Duodenum, Jejunum, Ileum, Cecum, Colon, Rectum

Animal number 02N10 Sex : Male Mode of death : Scheduled sacrifice Week(day) of death : 5 (29)
Test article : CNF「BiNFi-s」FMa-10002 Dose (mg/kg/day) : 400 Stage of death : Treatment period
Microscopic examination
The following organs were not remarkable: Stomach, Duodenum, Jejunum, Ileum, Cecum, Colon, Rectum
